# Multifunctional carbon nanotubes coated stainless steel mesh for electrowetting, hydrophobic, and dye absorption behavior

**DOI:** 10.1038/s41598-024-55087-5

**Published:** 2024-04-02

**Authors:** Hyeong Kwang Benno Park, Imen Kebaili, Imed Boukhris, Yun Hwan Joo, Tae Hyun Sung, Anuruddh Kumar

**Affiliations:** 1 Department of Electrical Engineering, Hanyang University, Seoul, 04763 South Korea; 2 Department of Physics, Faculty of Science, King Khalid University, P.O. Box 9004, Abha, Saudi Arabia; 3 Center for Creative Convergence Education, Hanyang University, Seoul, 04763 South Korea

**Keywords:** Hydrophobic, CNT, Electrowetting, Dye, Engineering, Materials science

## Abstract

Electrowetting behaviour for carbon nanotubes (CNT) grown on stainless steel mesh was investigated. The effect of temperature, time, and applied bias voltage on the contact angle of water droplets was studied. The impact of temperature variation on contact angle was also performed for the temperature ranging from 25 to 70 °C. A decrement of contact angle by 68% was observed for the mentioned range indicating a transition from a hydrophobic to hydrophilic nature. A similar trend was observed on the application of electric potential to the CNT-modified stainless-steel mesh ranging from 0 to 8 V with a transition of contact angle from 146 to 30 deg respectively. A comparative analysis for the contact angle variation with time for CNT-coated mesh and uncoated mesh was performed for 180 min. It is observed that uncoated mesh shows a reduction in contact angle to 0 deg with time while the CNT coated mesh shows surplus hydrophobicity with a 2 deg decrement in the extent of time. CNT-modified mesh successfully absorbs 95% of rhodamine B (RB) dye and detergent from water in 10 cycles.

## Introduction

Carbon-based materials have attracted wide attention due to their various applications including energy generation, energy storage, water cleaning or electrowetting, etc. Different allotropes of carbon including graphite, carbon black, graphene, and Carbon nanotubes (CNTs)^1–6^ show extensive properties in varied applications. CNTs are also known for their hydrophobic behaviour^7,8^. Superhydrophobic and hydrophilic surfaces have gained attraction in the last few decades. The dynamic real-time tuning of wetting properties has gained interest in a few years^9–11^. Controlling the wetting behavior of hydrophobic surfaces in the presence of an electric field is called electrowetting which involves the application of electric potential across solid liquid interface^12–18^. This in turn leads to the reduction in the contact angle at the liquid–solid interface by the application of an electric field. Electrowetting finds a huge application in various applications like electronic displays, variable focal length lenses, etc^.^^19–26^. Different carbon allotropes have shown electrowetting phenomena in particular CNT. Zhu et al.^27^ demonstrated the electrowetting phenomenon for the CNT arrays by transition from superhydrophobic behavior to hydrophilic behavior.

The literature does not present any evidence of the electrowetting phenomenon for the CNTs on conducting stainless steel substrates. In the present study, we initially grew CNTs on the stainless-steel mesh using the CVD technique followed by their electrowetting studies by voltage and time variations. Further, water cleaning is one of the grand challenges of this century. There are a variety of techniques available for water cleaning such as photocatalysis and adsorption. In order to degrade organic pollutants such as textile dyes, pharmaceutical waste, etc., photocatalysis is a known and widely explored technique. The photocatalysis technique is effective and driven by UV/ visible light. There is extensive literature available on carbon-based materials for water-cleaning applications such as absorption. Various authors have reported the absorption phenomenon on carbon nanotubes^21,22^. These switchable wetting properties of the CNTs can further be utilized in the removal of organic pollutants from water. In this study, we have analyzed the effect of the surface properties on the hydrophobicity with and without CNT-based surfaces on a stainless-steel mesh with time. The preset study deals with the study of CNT mesh for electrowetting application along with the absorption of rhodamine B dye and detergent.

## Experimental

A substrate with SS 304 grade wire mesh and 150 mesh per inch with a diameter of 50 µm was used for CNT growth using the CVD technique. The substrate was first cleaned by ultrasonication in acetone for 15 min followed by HCl for 30 min. This acid-treated mesh was utilized for CNT growth by loading in a CVD system. CNT growth was carried out on a stainless-steel mesh substrate in a quartz tube in a zone furnace CVD system on a stainless-steel mesh. Initially, a quartz tube with a 35 cm length in the heating zone is purged with the 700 sccm Ar for 5 min prior to CNT deposition. Followed by the growth of CNT at 700 °C with a preheating for 30 min at 850 °C using acetylene precursor. The precursor was used at a flow rate of 45 sccm for 5 min, leading to varying Ar flow rates at 300, 450, and 592 sccm for 15 min.

The morphology of the grown CNTs was analyzed using field emission scanning electron microscopy (FESEM). X-ray diffraction analysis was performed by Rigaku Powder diffractometer with scan speed of 2 degrees/min. Raman spectroscopy of CVD-grown CNTs was performed using Raman Horiba HR- Evolution spectrometer with laser wavelength 532 nm and 50× objective. A contact angle meter was used to measure the contact angle of a 10 µl droplet (Kyowa Interface Science Co. Ltd., Japan). The experimental setup was shown in Supplementary file 1. The absorption experiment was performed on rhodamine B (RB) dye and detergent. 20 ml volume of RB dye solution was taken in a petri dish. During the absorption experiments, RB dye and detergent solution of ~ 5 mg/L and 1 gm/L initial concentration was taken in a petri dish and CNT mesh sample were put into it.

## Results and discussion

**Figure 1:**
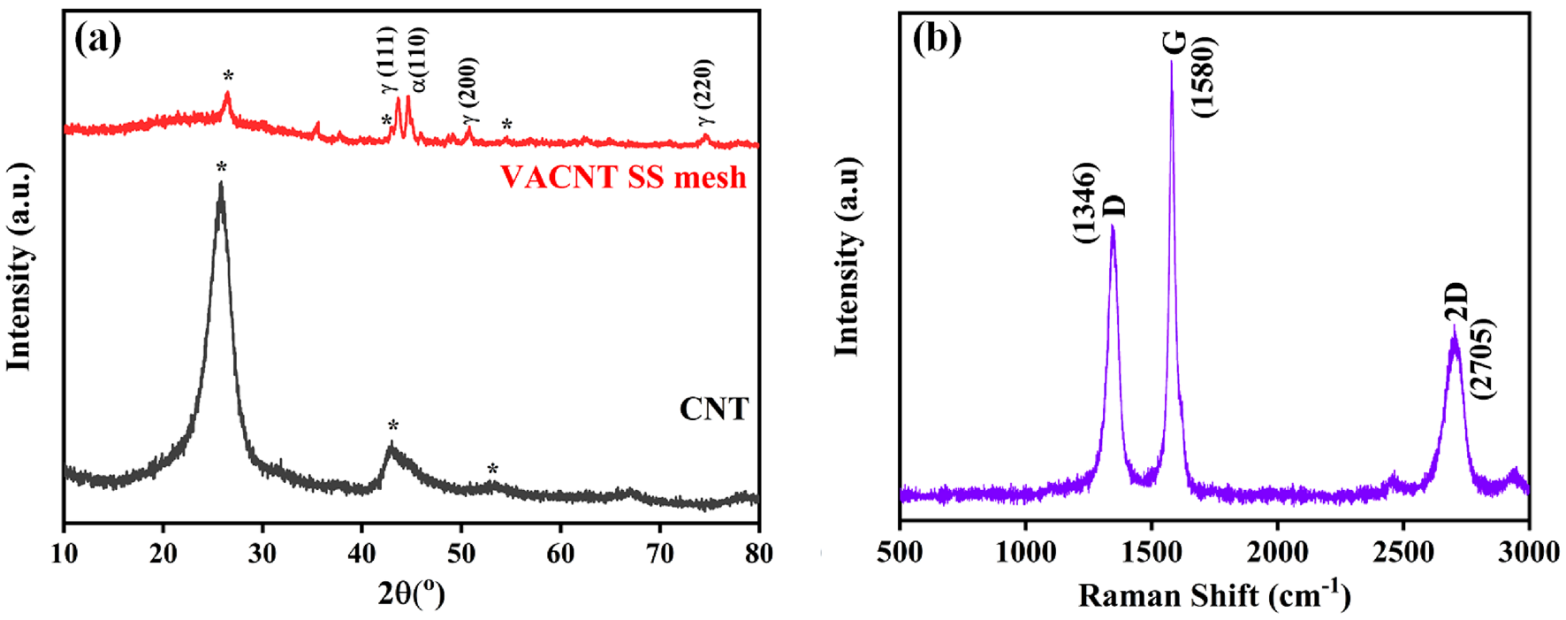
(**a**) XRD plots of CNT and VACNT on SS mesh, and (**b**) Raman spectrum of VACNT on SS mesh.

**Figure 2:**
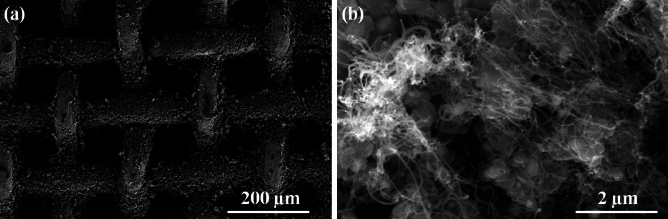
SEM images of VACNT growth on SS mesh at different magnifications.

Figure 1a shows the X-ray diffraction (XRD) plots of CNT powder and vertically aligned carbon nanotubes (VACNT) SS mesh. XRD plot of VACNT SS mesh shows the various diffraction peaks as γ-austenite, α-martensite, and carbon. VACNT SS mesh peaks are well matched with carbon nanotubes XRD peaks and stainless-steel peaks. OA Olaseinde et al. reported γ-austenite, α-martensite in stainless-steel^28–30^. Figure 1b shows the Raman spectrum of VACNT SS mesh at room temperature. In the VACNT SS mesh, the major peaks are at 1346 cm^−1^, 1580 cm^−1^, and 2705 cm^−1^. Vibrational Raman modes in the present case are well-matched with different hydrocarbon-related structures. The position of D and G bands was shown at 1346 cm^−1^, and 1580 cm^−1^. The ratio of the D and G bands (Id/Ig) was found to be 0.65. The ratio of D and G bands shows good structural quality of CNT growth. The 2D band was found at a position of 2704 cm^−1^. Figure 2 shows the scanning electron micrograph images of as-grown CNT from the stainless-steel mesh. CNT has grown homogeneously on the mesh.

**Figure 3:**
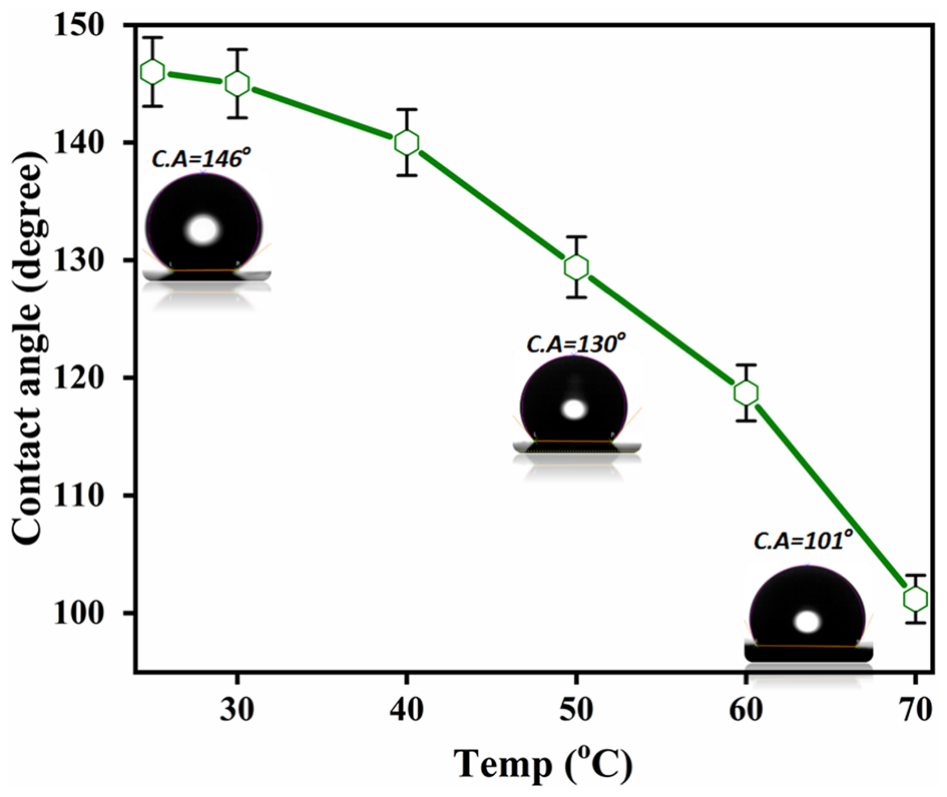
Variation in contact angle with temperature on VACNT mesh.

To check the effect of temperature on hydrophobicity of grown VACNT mesh. The contact angle is confirmed to be ~ 146 deg at room temperature (RT ~ 25 °C). However, a minor decrease in contact angle was seen with a rise in temperature to 50 °C, decreasing to ~ 130 deg. This reduction continued with a rise in temperature to 70 °C, decreasing to ~ 101 deg (Fig. 3). This indicates that a rise in temperature increases the hydrophilicity of the VACNT forest grown on SS mesh due to a decrement in the surface tension between solid and liquid surfaces^31–34^. This reduction in intermolecular forces thus leads to flattening of a liquid droplet on a higher temperature.

**Figure 4:**
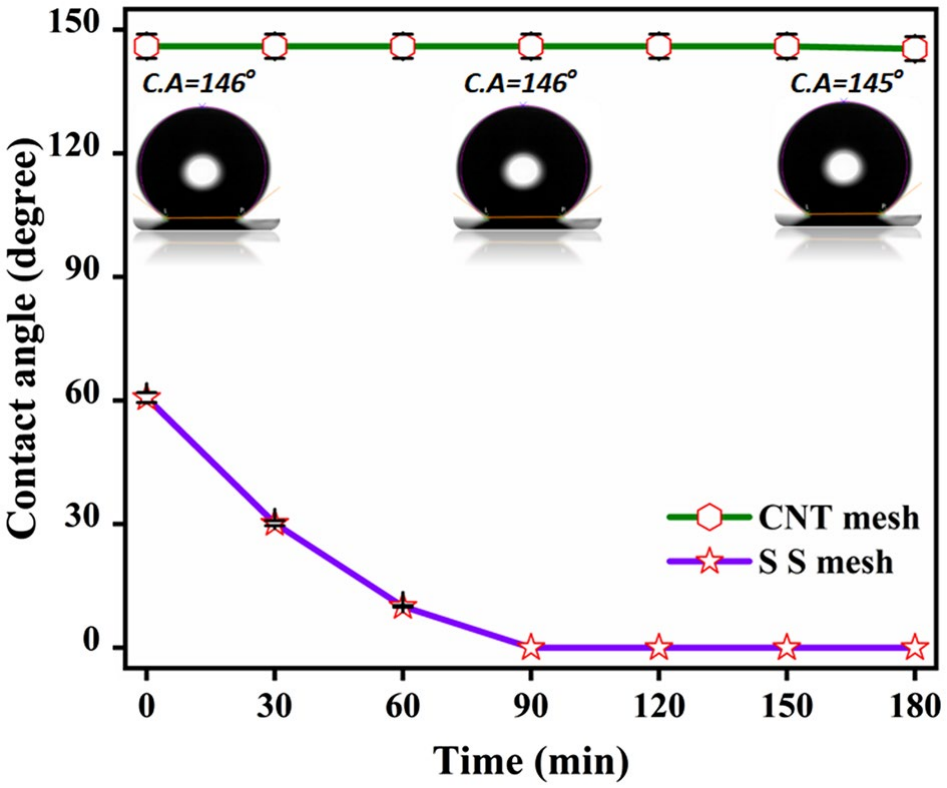
Time-dependent hydrophobic behavior.

A diversity in the contact angle with time was observed for SS and VACNT mesh. Figure 4 indicates that the uncoated mesh substrate exhibits hydrophilic nature and a contact angle of 60 deg was observed at t = 0 min which tremendously reduced to 0 deg at t = 90 min. On the other hand, the hydrophobic nature of VACNT mesh was confirmed by contact angle measurement. These results indicate a higher contact angle of ~ 146 deg at t = 0 min with a little reduction to 145 deg in 180 min of observation at RT. This difference of 145 deg in VACNT mesh w.r.t SS mesh indicates that a higher degree of hydrophobicity is achieved by surface modification of conventional SS mesh by CNTs. Such, hydrophobic steel meshes have many applications which include oil/ water separation, etc^35^.

**Figure 5:**
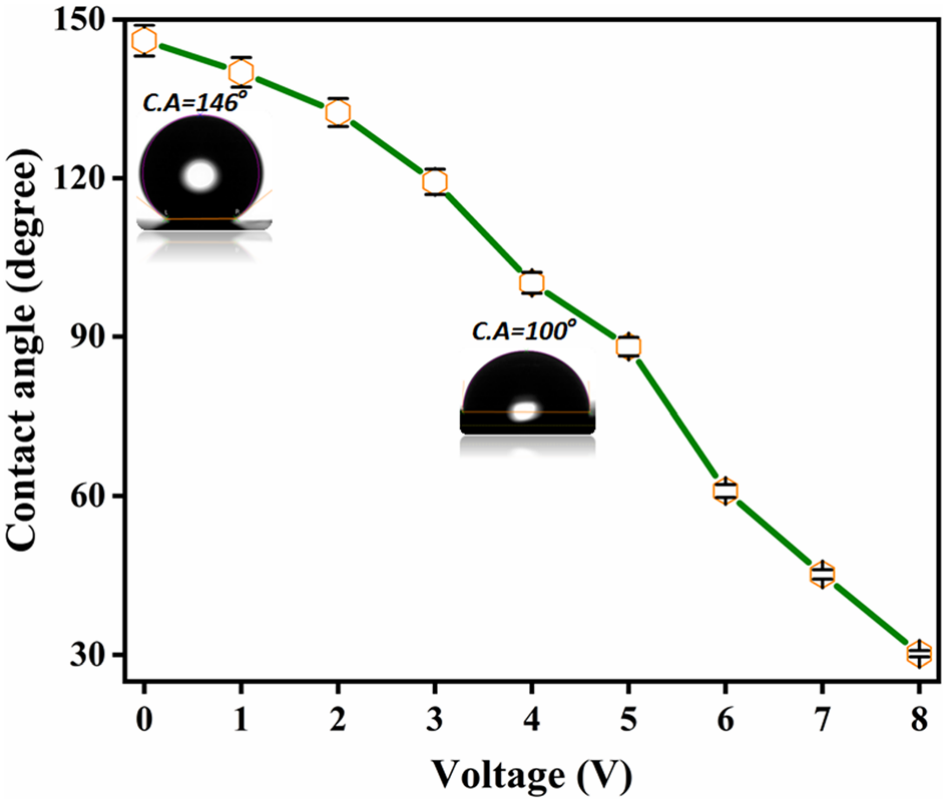
Variation in contact angle on the application of electric potential.

To further elucidate the electrowetting property of CNT-coated SS mesh, a corresponding test was performed. At 0 V potential contact angle of ~ 146 deg was observed which then reduced significantly to ~ 100 deg at the bias of 4 V and a large reduction to 30 deg was observed at a potential of 8 V as shown in Fig. 5. This fact thus satisfies the classical electrowetting Eq. (1) which signifies an increase in hydrophilicity with an increment in the applied bias. This is because, with the application of the applied bias, the interfacial tension between the solid and liquid droplet reduces; leading to flattening of the droplet^36^.

1 $$Cos\;\theta_{v} = Cos\;\theta_{0} + \frac{{\varepsilon \varepsilon_{0} }}{{2^{\prime}\Upsilon d}}V^{2}$$

where ${\theta }_{v}$: contact angle with the application of electric bias; ${\theta }_{0}$: contact angle without applied bias; V = applied bias; ${\varepsilon }_{0}$,ε is the permittivity of air and material respectively.

**Figure 6:**
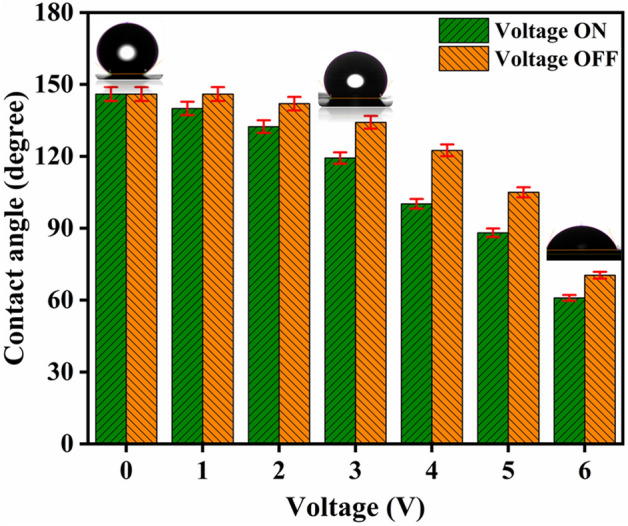
Reversible electrowetting at different voltages.

Thus, applied bias causes electric charges to flow onto the surface of the materials, saturation the contact angle. Measurements of contact angle bias between 0 and 6 V were taken at every location to validate the reversibility of electrowetting phenomena. If the contact angle recovers to its initial value after the applied voltage is removed, electrowetting is said to be reversible. Figure 6 shows that when the applied potential increases from 0 to 6 V, the contact angle decreases while returning to the same or higher angle after the applied field is removed. At 0 V, a contact angle of 146 deg is seen, which is the same as it would be in the absence of an applied electric field. The contact angle decreases to 140 deg at 1 V of potential bias before increasing back to 146 deg in the absence of bias. Similar trends were seen at various applied potentials, with the contact angle decreasing to a maximum of 60 deg at 6 V of applied voltage and increasing to 70 deg in the absence of bias. As a result, CNT mesh shows excellent electrowetting characteristics with reversible contact angles (Supplementary Fig. S1).

**Figure 7:**
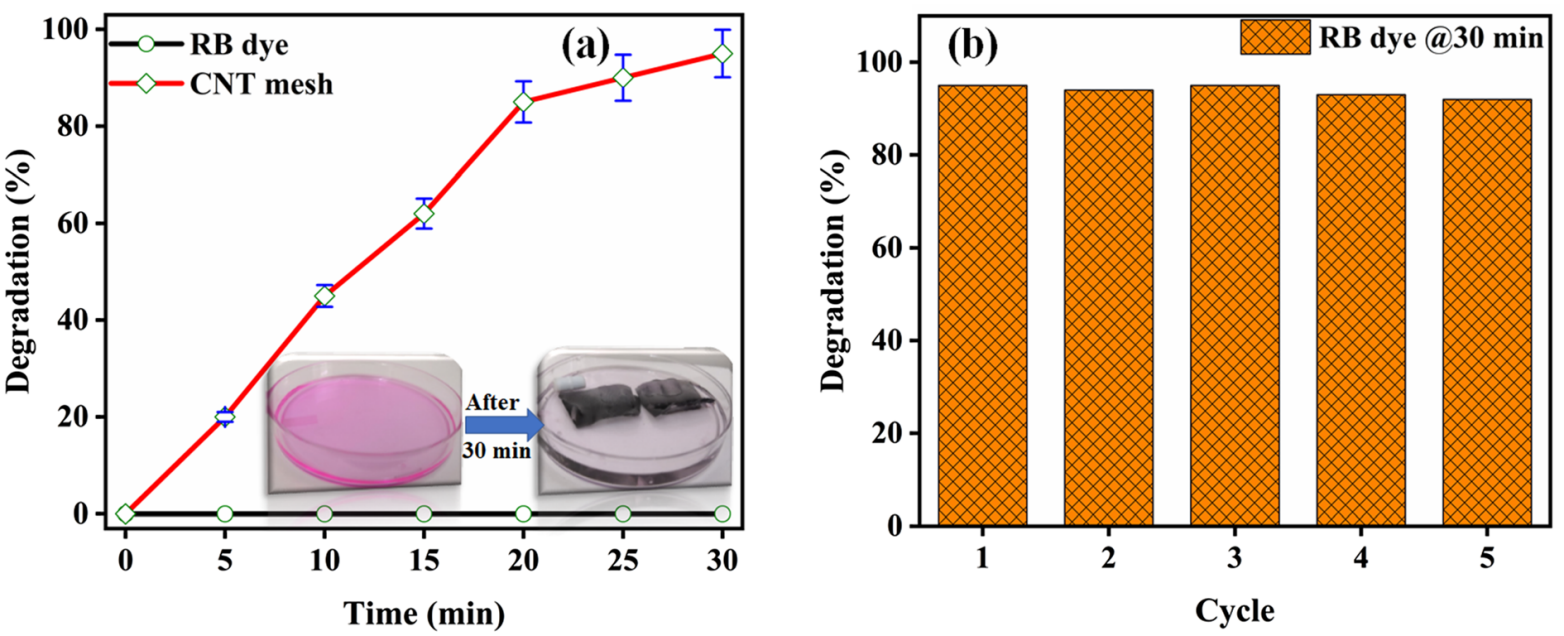
Removal of rhodamine B (RB) dye and reusability of CNT mesh.

Microporous structure and higher surface area of CNT forest on SS mesh leads to higher surface area. These assets of these materials can be used for the separation of contaminants from wastewater. To confirm this effect, we prepared a pink color solution of rhodamine dye (RB) in water as shown in Fig. 7a. CNT mesh was then placed in this pink RB-based water solution and the absorption of the dye process was analyzed with rest with reference to time. It has been found that the rate of absorption was very high up to 20 min due to the prescience of more active sites, this absorption was then reduced further and an absorption of ~ 95% of RB dye was confirmed in 30 min. Figure 7b shows the 95% degradation recorded in almost all 5 cycles of CNT mesh.

**Figure 8:**
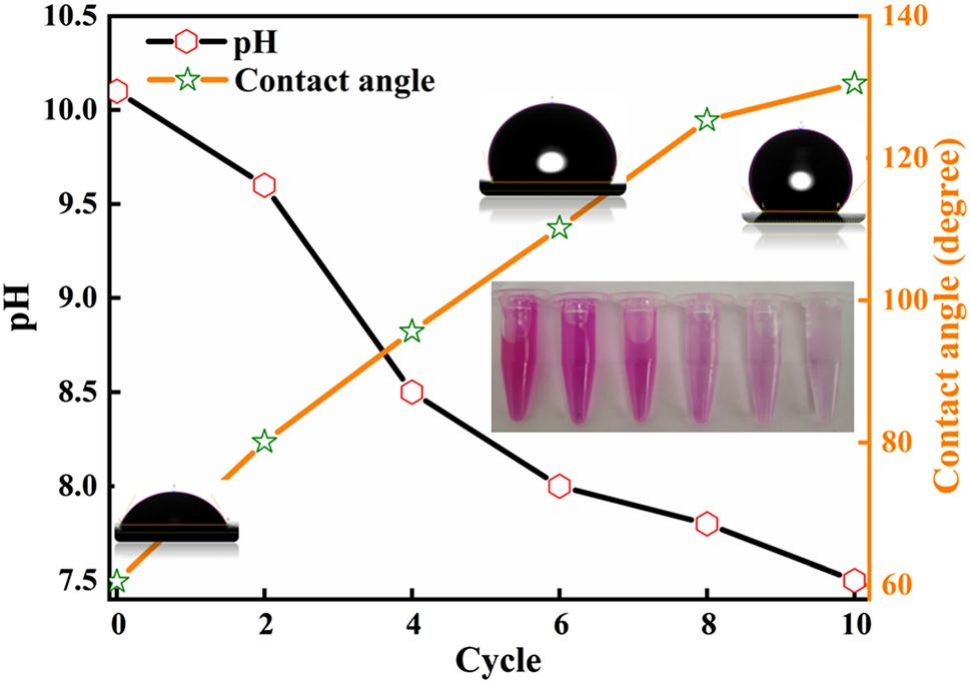
Variation in pH and hydrophobicity with treatment of detergent containing water.

As discussed earlier these structures are highly effective for absorbing contaminants from wastewater. Domestic wastewater is generally composed of various types of detergents which leads to contamination of both ground and surface water. Thus, there is a need for the successful removal of these surfactants during wastewater treatment. To analyze the eminence of CNT mesh in the removal of these surfactants, a detergent: water solution of 1 g/L was prepared. 20 ml of this solution was then stirred at 100 rpm with CNT mesh dipped inside with added phenolphthalein as a color indicator. The color transformation from pink to colorless indicates the removal of detergent from water. During the initial stage i.e., at 0 cycles, a dark pink color phenolphthalein solution was observed indicating the presence of a higher concentration of detergent. With the increase in no of cycles, the color transformation from dark pink to bright pink was observed indicating separation of detergent from solution. After 10 cycles a transparent solution was obtained indicating a large amount of detergent removal from the water. This was further confirmed by the pH and contact angle variation with a number of cycles. As presence of detergent shifts the pH of water to a higher level i.e. increases its basicity. So, at cycle 0 the pH of water was 10.2 with a lower contact angle of 60 deg. With the absorption of detergent from water the color of the solution changes with a reduction in pH level to 7.5 in 10 cycles and a contact angle of 130 deg as shown in Fig. 8. This thus confirms that as the detergent and other pollutants are removed from the treated wastewater, the contact angle of water to the CNT mesh increases. Hence CNT mesh both acts as an indicator and filter for the removal of pollutants from wastewater with a high degree of switchable wetting properties.

## Conclusions

Electrowetting of CNT-modified SS mesh was analyzed. The time-based study indicates the difference of 145 deg in the contact angle after 90 min suggesting better and prolonged hydrophobicity for CNT-modified SS mesh. The effect of applied potential from 0 to 8 V indicates a large decrement in the contact angle from 146 to 30 deg respectively due to a reduction in interfacial tension between the water droplet and CNT surface leading to an increment in hydrophilicity. A switchable transition in contact angle was observed with and without biasing conditions indicating better electrowetting properties on surface modification with CNT forest. This hydrophobic nature of CNT-modified mesh was then utilized for the eradication of detergent, the most common pollutant from domestic water using RB as an indicator. This was further confirmed using the variation in pH and contact angle with an increase in no. of cycles. pH was thus reduced from 10.2 to 7.5 with enhancement in hydrophobicity to contact angle of 130 deg after 10 cycles of treatment of RB containing detergent water.

### Supplementary Information

Supplementary Figure S1.

## Data Availability

The datasets used and/or analyzed during the current study are available from the corresponding author upon reasonable request.
