# Supplementary figures and images for: Multifunctional carbon nanotubes coated stainless steel mesh for electrowetting, hydrophobic, and dye absorption behavior
Source: Sci Rep. 2024 Apr 2;14:7738. doi: 10.1038/s41598-024-55087-5 (PMC10987552; doi:10.1038/s41598-024-55087-5)

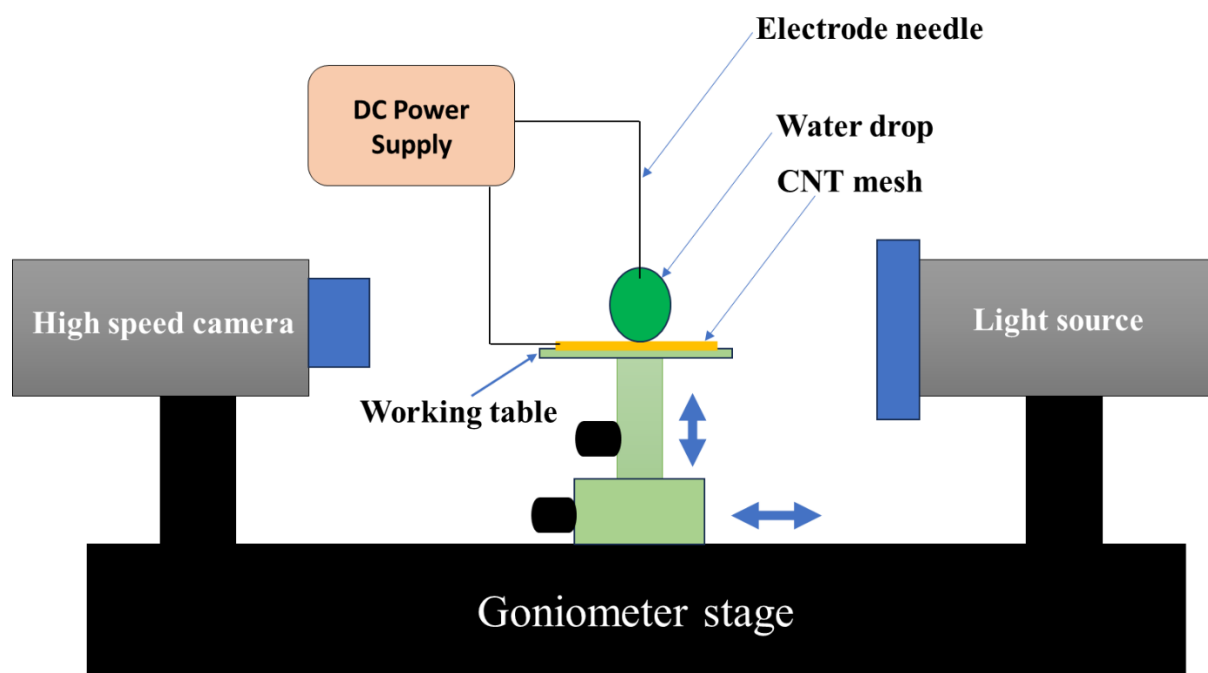

Figure S1: Electrowetting experimental setup

Supplement: Supplementary file 1 — Supplementary Figure S1. [file 41598_2024_55087_MOESM1_ESM.pdf]
